# Squamata reptiles as a potential source of helminth infections when preyed on by companion animals

**DOI:** 10.1186/s13071-023-05852-8

**Published:** 2023-07-14

**Authors:** Mariaelisa Carbonara, Jairo Alfonso Mendoza-Roldan, Riccardo Paolo Lia, Giada Annoscia, Roberta Iatta, Antonio Varcasia, Giuseppe Conte, Giovanni Benelli, Domenico Otranto

**Affiliations:** 1 Department of Veterinary Medicine, University of Bari, Valenzano, Bari, Italy; 2 Interdisciplinary Department of Medicine, University of Bari, Bari, Italy; 3 Department of Veterinary Medicine, University of Sassari, Sassari, Italy; 4 Department of Agriculture, Food and Environment, University of Pisa, Pisa, Italy; 5 Department of Pathobiology, Faculty of Veterinary Science, Bu-Ali Sina University, Hamedan, Iran

**Keywords:** Synanthropic reptiles, Helminth fauna, Predation by pets

## Abstract

**Background:**

Squamate reptiles cohabiting with companion animals may represent a source of helminth infections, especially through predation by dogs and cats with an outdoor lifestyle.

**Methods:**

In order to assess the role of reptiles as intermediate/paratenic hosts of trophically transmitted helminths, synanthropic reptiles (*n* = 245) captured from different ecological settings (i.e., households, dog shelters, urban, peri-urban and rural areas or natural parks) of southern Italy were examined for endoparasites. Parasitic cysts (i.e., larval forms of acanthocephalans, cestodes and nematodes) and free helminths (i.e., adult nematodes and digeneans) were morphologically and molecularly identified, and statistical analysis was carried out to evaluate the correlations between reptiles, infections, and ecological settings.

**Results:**

Overall, 31% of reptiles were positive for at least one helminth, with *Podarcis siculus* (18.7%) and *Tarentola mauritanica* (8.1%) being the most frequently infected species. Among the parasites of medical interest, *Joyeuxiella echinorhyncoides* showed the highest prevalence (19.7%), followed by *Diplopylidium acanthotetra* (10.5%), *Joyeuxiella pasqualei*, *Mesocestoides lineatus* (5.6%) and *Physaloptera* sp. (3.9%). *Macracanthorhynchus hirudinaceus* was detected once. *Podarcis siculus* and *T*. *mauritanica* were associated with cestode infections.

**Conclusions:**

The wide range of helminths detected here in reptiles living in sympatry with pets and the fact that many of these helminth species are parasitic and may infect companion animals (e.g.,* J*. *pasqualei*, *J. echinorhyncoides*, *D. acanthotetra*, *Physaloptera* sp.) and humans (i.e., *Macracanthorhynchus* *hirudinaceus*, *Mesocestoides* *lineatus*) indicate the potential health risk associated with pets preying on these small vertebrates. Our results indicate the need for complementary investigations of trophically transmitted parasites in dogs and cats living in sympatry with reptiles.

**Graphical Abstract:**

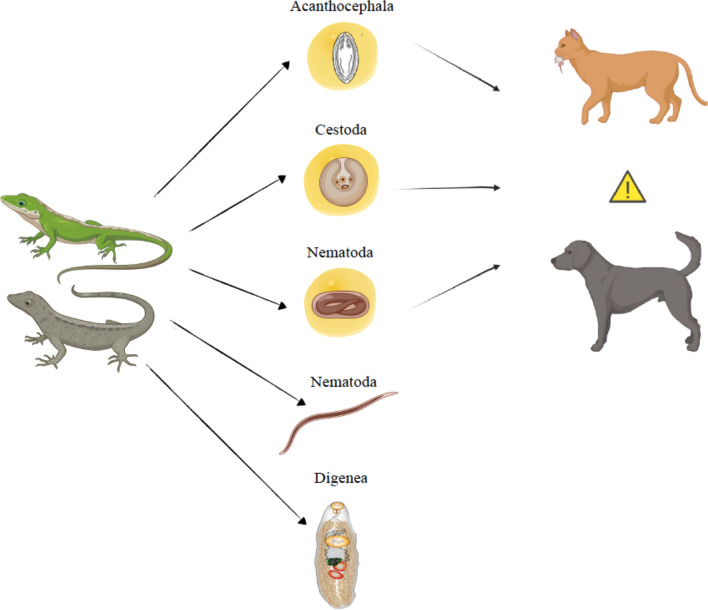

**Supplementary Information:**

The online version contains supplementary material available at 10.1186/s13071-023-05852-8.

## Background

Squamate reptiles, especially geckos and lizards, are common synanthropic animals with a worldwide distribution [1–3]. Urbanization and habitat fragmentation have favored the encounters between these animals, pets and humans, as well as the transmission of diseases caused by pathogens that are shared between them [3, 4]. Interactions between companion animals and reptiles have also increased due to the popularity of these small vertebrates as pets [5], with snakes and lizards being the most common [6]. Although information on the care of reptiles is still limited, they represent 2.1% of the pet population in Italy [7, 8]. As a result of their widespread popularity as pets, the scientific community’s interest in these animals has increased in recent decades, though little is known about their endoparasites [6]. Small reptiles preyed on by cats and dogs may represent a potential source of helminth infections, as some of these pathogens are trophically transmitted [9]. Indeed, many species of cestodes (i.e., *Joyeuxiella pasqualei*,* Joyeuxiella echinorhyncoides, Diplopylidium acanthotetra, Mesocestoides* spp.)*,* and nematodes (i.e., *Aelurostrongylus abstrusus, Physaloptera rara*, *Spirocerca lupi, Toxocara canis, Troglostrongylus brevior*) have reptiles as intermediate or paratenic hosts and pets as definitive ones [10–19]. Therefore, lizards and geckos cohabiting with companion animals represent a potential risk for the introduction of helminths into households, especially if these reptiles are obtained directly from the wild [20]. To date, helminths associated with reptiles have been investigated mainly from an ecological perspective [21–24], and only marginally in relation with their role in the maintenance of parasitic diseases of dogs and cats [9]. The main helminth taxa associated with synanthropic reptiles include Digenea (e.g., *Paradistomum* spp., *Brachylaima* spp.,* Renifer* spp.), Cestoda (e.g., *Diplopylidium* spp., *Joyeuxiella* spp. *Mesocestoides* spp.), Nematoda (e.g., oxyurids, ascarids, strongyles, *Rhabdias* spp., *Strongyloides* spp.) and Acanthocephala (e.g., *Sphaerirostris* spp., *Centrorhynchus* spp., *Oligacanthorhynchus* spp.) [24–29]. Cestodes and acanthocephalans are usually detected as larval forms, localized mostly in the coelomic cavity, liver and intestinal serosa. Digeneans and oxyurid nematodes, however, are generally detected as adults in the digestive tract [24, 26, 30, 31].

Although studies have been conducted on *Leishmania* spp., *Borrelia burgdorferi* and *Rickettsia* spp. associated with reptiles [32–34], there is still a lack of data on the endoparasites of these vertebrates. Thus, the aim of the present study was to evaluate the role of Squamata reptiles in different epidemiological settings (i.e., households, dog shelters, urban, peri-urban, and rural areas or natural parks) as sources of helminth infections when preyed on by companion animals.

## Methods

### Study area and reptile sampling

**Fig. 1:**
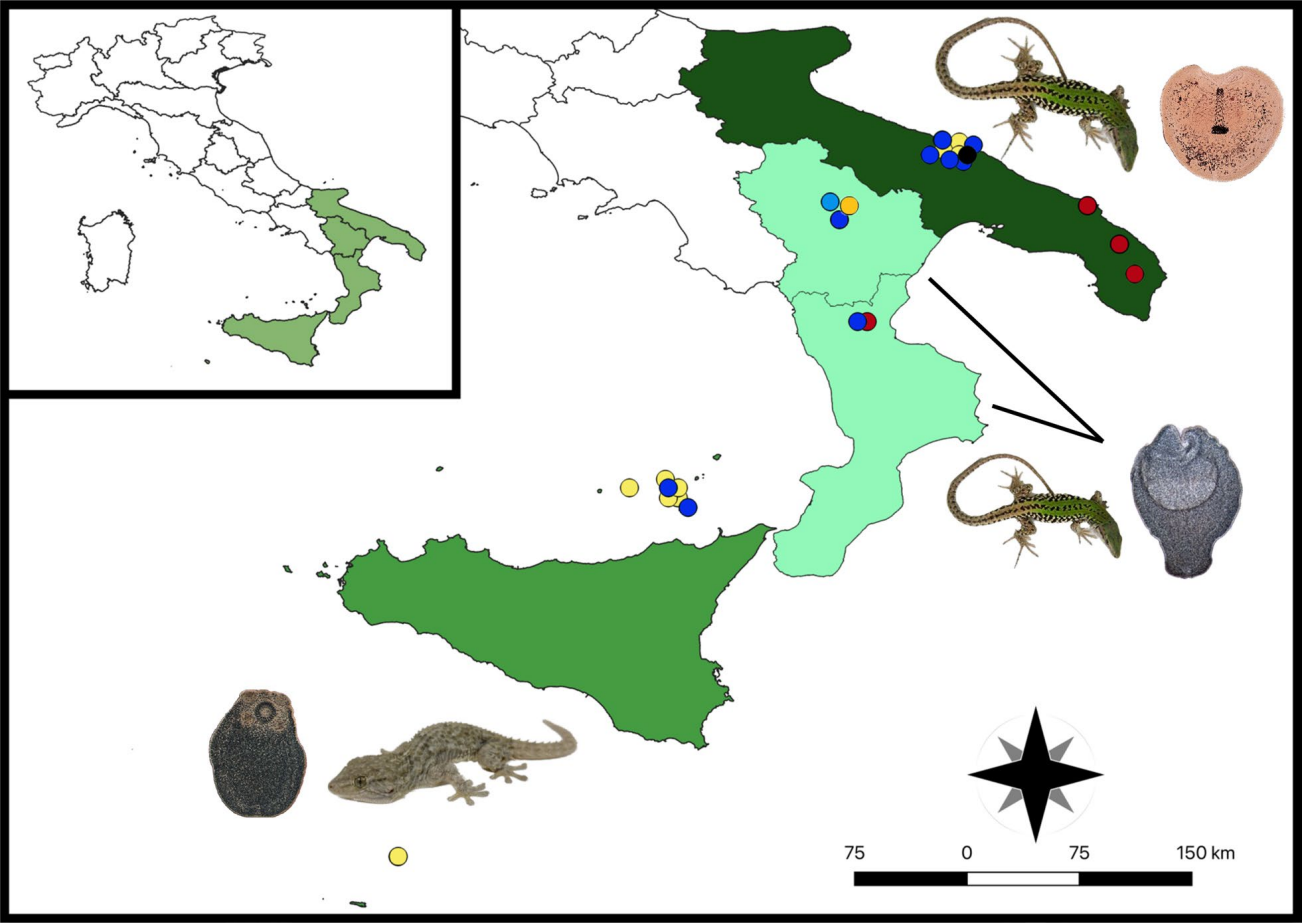
Map showing the sampling locations and the main parasite species of medical/veterinary interest and host species for the four Italian regions included in the study. Study locations/areas are represented by colored circles as follows: dog shelters (red circles), households (black circles), natural park (orange circle), peri-urban (blue circles), rural (light blue circle), urban (yellow circles). Shades of green indicate the abundance of collected hosts per region (e.g., darker green indicates higher abundance in the Apulia region). The most abundant species of parasite and host are indicated (i.e., *Joyeuxiella echinorhyncoides* and *Podarcis siculus* in Apulia, *Mesocestoides lineatus* and *Podarcis siculus *in Basilicata and Calabria, *Diplopylidium acanthotetra* and *Tarentola mauritanica* in Sicily)

Squamata reptiles were collected between April 2020 and July 2021 from various locations in four southern Italian regions (i.e., Apulia, Basilicata, Calabria, Sicily), within the framework of a study on zoonotic parasites of reptiles [34]. Specifically, the study locations (i.e., households, dog shelters, urban, peri-urban, and rural areas or natural parks) of each region were chosen based on the presence of different reptile species living in sympatry with the feline, canine and human population (Fig. 1). Data on the reptiles, including geographical origin (i.e., region, city/town, study location), capture status (i.e., dead or alive) and biological status (i.e., young, young adult, adult) were recorded in individual files, along with information on the presence of companion animals at the same location, and antiparasitic treatments (i.e., insecticides/repellents, anthelmintics). Two hundred and thirty of the captured reptiles were humanely euthanized according to protocols [35]. Necropsies were performed on these and on the reptiles captured dead (*n* = 15). The body wall was opened by longitudinal incision and the coelomic cavity, organ surfaces, mesenteries and organ lumens examined for the presence of helminths by optical observation using a stereomicroscope (Leica MS5; Leica, Germany). Protocols for the collection of reptiles were authorized by the Ministry for Environment, Land and Sea Protection of Italy (approval no. 0073267/2019).

### Morphological identification of helminths

**Fig. 2:**
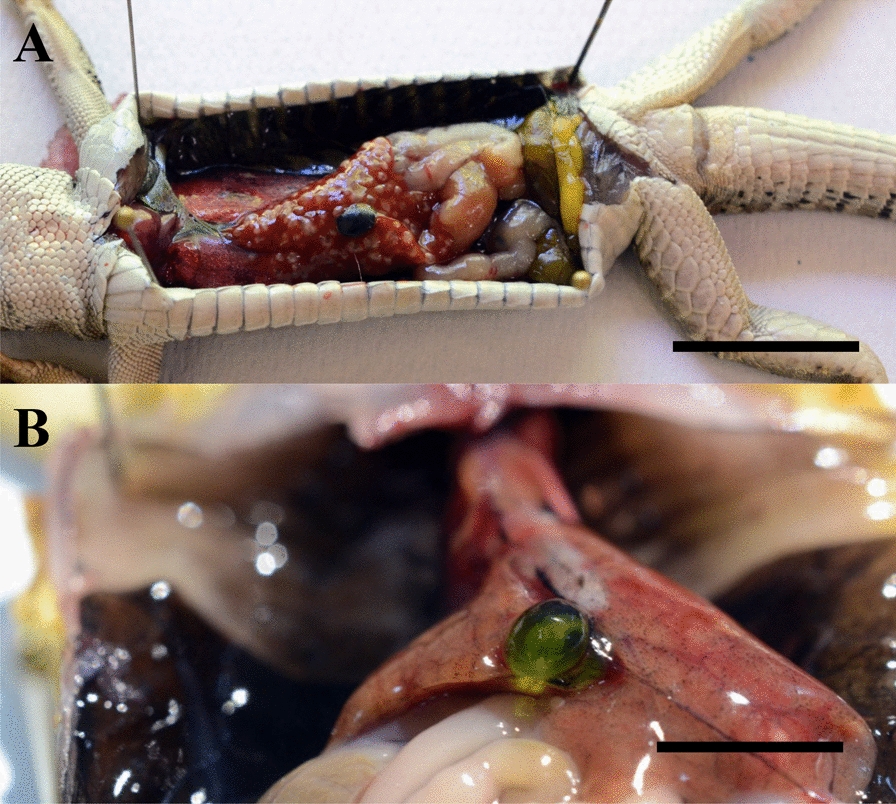
Ventral view of dissected reptiles showing (**a**) liver cysts (scale bar = 4 cm), and (**b**) gall bladder with digenean (scale bar = 1 cm)

Parasitic cysts were separated from non-parasitic ones and examined under a light microscope (Leica DMLB2), as were free helminths (Fig. 2). A representative number of individuals of each parasite group (i.e., Acanthocephala, Nematoda, Cestoda, and Digenea) was fixed and cleared on a glass slide. For cestodes, digeneans and acanthocephalans, formalin/acetic acid/alcohol solution was used, while nematodes were cleared in lactophenol solution and examined as temporary preparations. All of the remaining cysts and/or free helminths collected were stored in individual vials containing 70% ethanol. After storage for a few hours (for nematodes) or a maximum of 2 days (for other helminths) at 25 °C, the slides were examined using light microscopy; dichotomous keys and original descriptions were used for morphological identification [16, 31, 36–56]

### Molecular procedures

For the molecular identification of helminths, genomic DNA was extracted using a commercial kit (Qiagen Micro Kit; Qiagen, Hilden, Germany) in accordance with the manufacturer’s instructions, for a representative number of specimens. At least one sample (or more than one in the case of different host species and/or regions of collection) for each parasite of veterinary and medical concern was processed, along with three acanthocephalan specimens, as these were the most common helminth group detected. The quantity of the DNA of eight samples was evaluated by Qubit 2.0 fluorometer (Applied Biosystems, USA). Conventional polymerase chain reaction (PCR) was performed for molecular identification of acanthocephalans as well as for cestodes and nematodes; details regarding sample processing, including the target genes and primers used, are reported in Table 1. Amplified PCR products were visualized by gel electrophoresis in 2% agarose gel containing GelRed nucleic acid gel stain (VWR International, Milan, Italy) and viewed on a GelLogic 100 gel documentation system (Kodak, New York). Negative (i.e., ultra-pure sterile water) and positive DNA controls collected in previous studies (i.e., *Sphaerirostris picae*, *J*. *pasqualei*,* T*. *brevior*) were included in all PCR runs. All the positive PCR products were purified and sequenced in both directions using the same forward and reverse primers by employing Big Dye Terminator v.3.1 chemistry (3130 Genetic Analyzer; Applied Biosystems, CA) in an automated sequencer (ABI-PRISM 377). Nucleotide sequences were edited, aligned and analyzed using Geneious version 9.0 (Biomatters, Auckland, New Zealand) [57], and compared with publicly available sequences in the GenBank database, using the Basic Local Alignment Search Tool (BLAST; http://blast.ncbi.nlm.nih.gov/Blast.cgi) for species identification.

**Table 1:** Molecular tools employed for helminth identification

Helminth	Target gene	Primer	Sequence 5′-3′	Annealing temperature (°C)	Fragment length (base pairs)	References
*Macracanthorhynchus hirudinaceus*	*cox*1	JB3	TTTTTTGGGCATCCTGAGGTTAT	48	~ 400	[89]
*Diplopylidium acanthotetra*						
*Joyeuxiella* *echinorhyncoides*		JB4.5	TAAAGAAAGAACATAATGAAAATG			
*Mesocestoides lineatus*						
*Sphaerirostris picae*		Cyclo* cox*1Fa	CARCATATGTTTTGRTTTTTTGG	52	~ 420	[49, 90]
*Diplopylidium acanthotetra*						
*Joyeuxiella* *echinorhyncoides*		Cyclo* cox*1Rb	CCTAAYGACATAACATAATGRAAATG			
*Physaloptera* sp.	*cox*1	NTF	TGATTGGTGGTTTTGGTAA	54	~ 555	[91]
		NTR	ATAAGTACGAGTATCAATATC			
	18S	NC18SF	AAAGATTAAGCCATGCA	57	~ 1700	[92]
		NC5BR	GCAGGTTCACCTACAGAT			
		Worm AF	GCGAATGGCTCATTAAATCAG	54	~ 530	[93]
		1270R	CCGTCAATTCCTTTAAGTTT			

### Statistical analysis

For reptile species from various ecological and environmental settings that were or were not infected by helminth parasites, a multiple correspondence analysis (MCA) was carried out to graphically represent the relationship structure of two or more qualitative variables through positioning maps [58]. Before conducting the MCA analyses, several responses were transformed into dichotomous variables (yes/no) [58]. The suitability of the MCA variables was assessed by chi-squared tests. Variables showing significant correlations with less than half of the total variables were discarded. Eigen values that were more than the value of the mean [59] and Cronbach’s alpha [60] were used for the selection of an appropriate number of dimensions. The MCA analysis conducted here was undertaken to assess the potential relevance of selected ecological, epidemiological, and environmental variables with respect to the health risk of companion animals linked to potential reptile predation. Eleven variables were included in the analysis: reptile capture status (dead or alive); region (Apulia, Basilicata, Calabria, and Sicily); type of sampling location (urban, peri-urban, rural, regional park, household and dog shelter); presence of pets (yes/no); treatment of pets with insecticides/repellents (yes/no); treatment of pets with anthelmintics (yes/no); presence of cysts (yes/no); positivity of the examined reptiles with respect to Acanthocephala (yes/no), Nematoda (yes/no), Cestoda (yes/no), Digenea (yes/no). Exact binomial 95% confidence intervals (CIs) were established for proportions by using Epitools—Epidemiological Calculators software [61].

## Results

A total of 245 squamate reptiles belonging to five families (i.e., Scincidae, Gekkonidae, Lacertidae, Pythonidae and Phyllodactylidae) and seven species (see Table 2), of which 75.1% were classified as adults and the remaining as young adults (15.9%) or young (8.9%), were subjected to necropsy. The species of reptiles captured are reported in Table 2 together with geographical location (i.e., sampling location, city/town, region) and capture status (dead or alive).

**Table 2:** Reptile species and number of individuals captured with respect to sampling location, capture status and presence of at least one cyst and/or one helminth

Species (no.)	Region (no.)	City/town (no.)	Sampling location (no.)	Capture status (no.)	Cysts (no.)	Helminths (no.)
*Chalcides ocellatus* (25)	Sicily (25)	Linosa (25)	Urban (25)	Alive (25)	4	6
*Hemidactylus turcicus* (2)	Calabria (2)	Cassano All'ionio (2)	Dog shelter (2)	Alive (2)	0	0
*Hierophis carbonarius* (3)	Sicily (1)	Salina (1)	Urban (1)	Alive (1)	1	1
	Apulia (2)	Torre a Mare (1)	Urban (1)	Dead (1)	1	1
		Noicattaro (1)	Urban (1)	Dead (1)	0	0
*Podarcis filfolensis* (22)	Sicily (22)	Linosa (22)	Urban (22)	Alive (22)	1	2
*Podarcis siculus* (125)	Apulia (81)	Brindisi (17)	Dog shelter (17)	Alive (17)	6	6
		Lecce (30)	Dog shelter (30)	Alive (30)	21	16
		Noicattaro (15)	Household (9)	Dead (1)	0	0
				Alive (8)	6	4
			Urban (4)	Dead (4)	3	0
			Peri-urban (2)	Dead (2)	0	0
		Torre a Mare (3)	Peri-urban (2)	Alive (2)	2	2
			Urban (1)	Dead (1)	0	0
		Valenzano (16)	Peri-urban (16)	Dead (1)	1	1
				Alive (15)	11	5
	Basilicata (10)	Parco regionale (10)	Natural park (10)	Alive (10)	6	4
	Calabria (17)	All'ionio (17)	Dog shelter (12)	Alive (12)	7	3
			Peri-urban (5)	Alive (5)	2	1
	Sicily (17)	Filicudi (1)	Urban (1)	Alive (1)	1	1
		Linosa (1)	Urban (1)	Alive (1)	0	0
		Lipari (9)	Urban (9)	Alive (9)	2	3
		Malfa (4)	Peri-urban (4)	Alive (4)	0	0
		Vulcano (2)	Peri-urban (2)	Alive (2)	0	0
*Python molurus* (1)	Apulia (1)	Valenzano (1)	Urban (1)	Dead (1)	0	0
*Tarentola mauritanica* (67)	Apulia (25)	Adelfia (1)	Peri-urban (1)	Dead (1)	1	0
		Bari (6)	Peri-urban (6)	Alive (6)	5	5
		Brindisi (1)	Dog shelter (1)	Alive (1)	0	0
		Lecce (3)	Dog shelter (3)	Alive (3)	1	1
		Noicattaro (6)	Household (3)	Alive (3)	2	2
			Urban (2)	Alive (2)	2	0
			Peri-urban (1)	Dead (1)	0	0
		Tricase (1)	Dog shelter (1)	Alive (1)	1	1
		Valenzano (7)	Peri-urban (7)	Alive (7)	6	4
	Basilicata (1)	Pietrapertosa (1)	Rural (1)	Alive (1)	0	0
	Calabria (5)	Cassano All'ionio (5)	Dog shelter (5)	Alive (5)	0	0
	Sicily (36)	Linosa (22)	Urban (22)	Alive (22)	3	2
		Lipari (4)	Urban (4)	Alive (3)	2	2
				Dead (1)	0	0
		Malfa (8)	Peri-urban (6)	Alive (6)	3	2
			Urban (2)	Alive (2)	2	1
		Pollara (2)	Urban (2)	Alive (2)	0	0
Total 245					103	76

Of the 245 reptiles examined, 42% had at least one cyst (103/245, 95% CI 0.36–0.48) (Table 2). Of these, 62.1% (64/103, 95% CI 0.52–0.71) had cysts containing parasite larval forms. Specifically, 13% (32/245, 95% CI 0.09–0.18) of the animals were infected with acanthocephalans, 12.6% (31/245, 95% CI 0.09–0.17) with cestodes, and 4% (10/245, 95% CI 0.02–0.07) with nematodes. In addition, 9.7% of the reptiles examined (i.e., 24/245, 95% CI 0.07–0.14) had at least one free helminth, and 6.1% (15/245, 95% CI 0.04–0.10) were parasitized by adult nematodes and 3.6% (9/245, 95% CI 0.02–0.07) by digeneans, which were localized in the intestinal lumen and gall bladder, respectively. Overall, 31% of the reptiles (76/245, 95% CI 0.26–0.37) were positive for at least one helminth (Table 2) and 22.3% (17/76, 95% CI 0.14–0.33) of them were co-infected with two or three parasite groups, with Acanthocephala-Nematoda the most common association recorded (Table 3). *Podarcis siculus* (18.7%, 46/245, 95% CI 0.14–0.24) and *T. mauritanica* (8.1%, 20/245, 95% CI 0.05–0.12) were the species most frequently found to be positive for parasites (Table 2). All of the infected reptiles lived in sympatry with pets, of which 43.4% had been previously treated with repellent/insecticide and 7.9% also with anthelmintics.

**Table 3:** Reptile species infected by more than one parasite group [Acanthocephala (*A*), Cestoda (*C*), Nematoda (*N*), Digenea (*D*)], and location from which each individual was collected

Species	City/town	Region	Sampling location	Parasite group
*Tarentola mauritanica*	Noicattaro	Apulia	Household	C + D
*Podarcis siculus*	Lecce	Apulia	Dog shelter	A + N
*Podarcis siculus*	Lecce	Apulia	Dog shelter	A + N
*Podarcis siculus*	Lecce	Apulia	Dog shelter	A + N
*Tarentola mauritanica*	Noicattaro	Apulia	Household	N + C
*Podarcis siculus*	Valenzano	Apulia	Peri-urban	A + N + D
*Tarentola mauritanica*	Valenzano	Apulia	Peri-urban	A + N + C
*Podarcis siculus*	Valenzano	Apulia	Peri-urban	A + C
*Podarcis siculus*	Torre a mare	Apulia	Peri-urban	A + C + D
*Podarcis siculus*	Brindisi	Apulia	Dog shelter	A + D
*Podarcis siculus*	Lecce	Apulia	Dog shelter	N + C
*Podarcis siculus*	Lecce	Apulia	Dog shelter	A + N
*Chalcides ocellatus*	Linosa	Sicily	Urban	A + N + D
*Podarcis filfolensis*	Linosa	Sicily	Urban	A + C
*Podarcis siculus*	Valenzano	Apulia	Peri-urban	C + D
*Podarcis siculus*	Cassano All'ionio	Calabria	Peri-urban	A + C
*Tarentola mauritanica*	Valenzano	Apulia	Peri-urban	A + C

A total of 12 parasite taxa were morphologically identified, including larvae of two acanthocephalans (*S. picae*, *Macracanthorhynchus hirudinaceus*) and four cestodes (*D*. *acanthotetra*, *J*. *pasqualei*, *J*. *echinorhyncoides*, *Mesocestoides* *lineatus*), third-stage larvae of two nematodes (family Acuariidae, *Physaloptera* sp.), adults of three nematodes (*Parapharygodon micipsae*, *Spauligodon aloisei*, *Moaciria icosiensis*), and an adult of one digenean (*Paradistomum mutabile*). The main morphological features allowing macroscopic identification, along with morphometric measurements and photos, are provided in additional files (Additional file 1: Text S1, Figure S1, S2, S3, S4, S5, S6, S7, S8, S9, S10, S11, S12, Table S1).

In the BLAST analysis, the *cox*1 sequences of *S*. *picae* (Cyclo cox1Fa/Cyclo cox1Rb) and *M*. *lineatus* (JB3/JB4.5) shared 99.7% and 98.9% nucleotide identity with GenBank sequences MK471355 and JF268501, respectively. The 18S nucleotide sequence of *Physaloptera* sp. (worm AF/1270R) was 99.4% similar to sequence MN855524. For the other parasite taxa investigated by molecular methods (i.e., *D*. *acanthotetra*, *J*. *echinorhyncoides*, *M*. *hirudinaceus*) no amplification was recorded, or the sequences were of low quality; the sequence of *J*. *pasqualei* was previously reported [54].

Of the helminth parasites of medical interest identified in the reptiles (*n* = 76), occurrence was highest for *J*. *echinorhyncoides* (19.7%, 15/76, 95% CI 0.12–0.30), followed by *D*. *acanthotetra* (10.5%, 8/76, 95% CI 0.05–0.19), *J*. *pasqualei* and *M*. *lineatus* (5.6%, 4/76, 95% CI 0.02–0.13), and *Physaloptera* sp. (3.9%, 3/76, 95% CI 0.01–0.11). *Macracanthorhynchus hirudinaceus* was detected in one *P*. *siculus* specimen, which was also infected with *S*. *picae*. Information on the identified parasites, including taxa, helminth stage, anatomical site, host species and sampling locations, is given in Table 4. Among the infected *P*. *siculus* specimens (*n* = 46), *S*. *picae* (43.4%, 20/46, 95% CI 0.30–0.58) and *J*. *echinorhyncoides* (23.9%, 11/46, 95% CI 0.14–0.38) were the most frequent helminths detected, followed by nematodes of the family Acuariidae (13%, 6/46, 95% CI 0.06–0.26). The most frequently recorded association was that of *S*. *picae* with nematodes of the family Acuariidae (Tables 2, 3, 4). Of the 20 *T. mauritanica* positive on necropsy, occurrence was highest for *S*. *picae* (40%, 8/20, 95% CI 0.22–0.61), followed by *D*. *acanthotetra* (25%, 5/20, 95% CI 0.11–0.47) (Tables 2, 3, 4).

**Table 4:** Taxa and stage of identified parasitic helminths, anatomical site [liver (*L*), intestinal serosa (*IS*), mesentery (*M*), peritoneum (*P*), intestinal lumen (*IL*), stomach (*S*), gall bladder (*G*)], host species and sampling location

Helminth taxa	Stage	Anatomical site	Host (no.)	Region (no.)	City/town (no.)	Sampling location
Acanthocephala						
* Sphaerirostris picae*	Larval	L, IS, M, P	*Chalcides ocellatus* (1)	Sicily (1)	Linosa (1)	Urban
			*Podarcis filfolensis* (1)	Sicily (1)	Linosa (1)	Urban
			*Podarcis siculus* (22)	Calabria (1)	Cassano All' ionio (1)	Peri-urban
				Apulia (20)	Brindisi (5)	Dog shelter
					Lecce (8)	Dog shelter
					Noicattaro (2)	Household
					Torre a mare (1)	Peri-urban
					Valenzano (4)	Peri-urban
				Sicily (1)	Filicudi (1)	Urban
			*Tarentola mauritanica* (8)	Apulia (8)	Bari (3)	Peri-urban
					Lecce (1)	Dog shelter
					Tricase (1)	Dog shelter
					Valenzano (3)	Peri-urban
* Macracanthorhynchus hirudinaceus*	Larval	L	*Podarcis siculus* (1)	Apulia (1)	Valenzano (1)	Peri-urban
Nematoda						
* Acuariidae* gen. sp.	Larval	IS, P	*Chalcides ocellatus* (1)	Sicily (1)	Linosa (1)	Urban
			*Podarcis siculus* (5)	Apulia (5)	Lecce (4)	Dog shelter
					Brindisi (1)	Dog shelter
			*Tarentola mauritanica* (1)	Apulia (1)	Valenzano (1)	Peri-urban
* Physaloptera* sp.	Larval	IS, S	*Hierophis carbonarius* (1)	Sicily (1)	Salina (1)	Urban
			*Podarcis siculus* (2)	Apulia (1)	Valenzano (1)	Peri-urban
				Calabria (1)	Cassano All' ionio (1)	Peri-urban
* Parapharygodon micipsae*	Adult	IL	*Tarentola mauritanica* (5)	Sicily (4)	Lipari (3)	Urban
					Linosa (1)	Urban
				Apulia (1)	Noicattaro (1)	Household
* Spauligodon aloisei*	Adult	IL	*Chalcides ocellatus* (1)	Sicily (1)	Linosa (1)	Urban
			*Podarcis filfolensis* (2)	Sicily (2)	Linosa (1)	Urban
			*Podarcis siculus* (3)	Sicily (1)	Lipari (1)	Urban
				Basilicata (1)	Regional park (1)	Natural park
				Apulia (1)	Lecce (1)	Dog shelter
* Moaciria icosiensis*	Adult	IL	*Chalcides ocellatus* (4)	Sicily (4)	Linosa (4)	Urban
Cestoda						
* Diplopylidium acanthotetra*	Larval	L, IS	*Podarcis siculus* (3)	Apulia (2)	Noicattaro (2)	Household
				Calabria (1)	Cassano All' ionio (1)	Dog shelter
			*Tarentola mauritanica* (6)	Apulia (3)	Noicattaro (2)	Household
					Valenzano (1)	Peri-urban
				Sicily (3)	Malfa (3)	Urban
						Peri-urban
* Joyeuxiella pasqualei*	Larval	L	*Podarcis siculus* (3)	Apulia (2)	Lecce (2)	Dog shelter
				Basilicata (1)	Regional park (1)	Natural park
			*Tarentola mauritanica* (1)	Apulia (1)	Noicattaro (1)	Household
* Joyeuxiella echinorhyncoides*	Larval	L, IS	*Hierophis carbonarius* (1)	Apulia (1)	Torre a Mare (1)	Urban
			*Podarcis siculus* (11)	Apulia (10)	Lecce (6)	Dog shelter
					Torre a Mare (1)	Peri-urban
					Valenzano (3)	Peri-urban
				Sicily (1)	Lipari (1)	Urban
			*Tarentola mauritanica* (3)	Apulia (3)	Bari (1)	Peri-urban
					Valenzano (2)	Peri-urban
* M. lineatus*	Larval	L	*Podarcis siculus* (4)	Basilicata (2)	Regional park (2)	Natural park
				Calabria (2)	Cassano All' ionio (2)	Dog shelter
Digenea						
* P. mutabile*	Adult	G	*Chalcides ocellatus* (1)	Sicily (1)	Linosa (1)	Urban
			*Podarcis siculus* (6)	Apulia (5)	Brindisi (1)	Dog shelter
					Valenzano (2)	Peri-urban
					Torre a Mare (2)	Peri-urban
				Sicily (1)	Lipari	Urban
			*Tarentola mauritanica* (2)	Apulia (2)	Noicattaro (1)	Household
					Bari (1)	Peri-urban

**Fig. 3:**
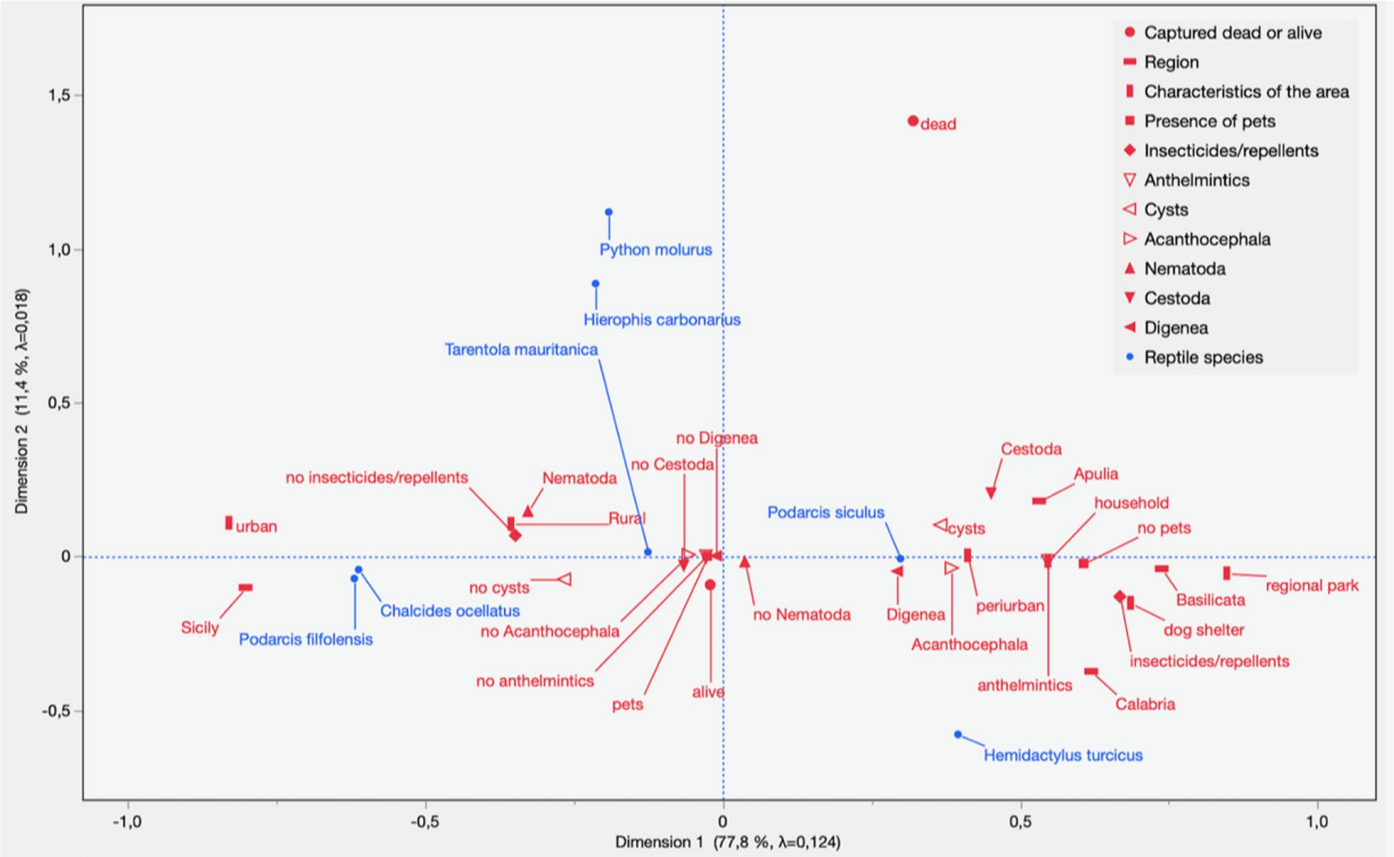
Multiple correspondence analysis biplot for reptile species infected or not infected with helminth parasites in various ecological, epidemiological, and environmental settings

The MCA analysis conducted using the 11 variables identified two dimensions which explained 89% of the variability, with a Cronbach’s α of 0.406 (Fig. 3). The latter is related to a weighted average of the correlations between the variables in the MCA, and is used to assess the overall reliability of the new measurement scale, created using the extracted dimensions, so as to ascertain if the obtained value that is retained is acceptable [58, 62]. Dimension 1 was associated with region (eigenvector > 0.6—Basilicata 0.738, Calabria 0.619, Sicily − 0.802), study location (dog shelter 0.686, regional park 0.847, urban − 0.830), pet presence (no pets 0.607), use of insecticides/repellents (yes 0.668), and explained 77.8% of the variability (Fig. 3) (Additional file 2: Table S2). Dimension 2 was associated with status when captured (dead 1.417) and explained 11.4% of the variability (Additional file 2: Table S2). Chi-square test results, inertia and the explained percentage variability for each singular value are given in Table 5. Figure 3 shows that *P*. *siculus* was associated with Apulia, the presence of cysts and Cestoda, while *T*. *mauritanica* was associated with urban environments, and to a minor extent, the presence of Nematoda and pets not treated with insecticides or repellents.

**Table 5:** Multiple correspondence analysis showing chi-square test results, inertia and percentage explained variability for each singular value

Singular value	Inertia	χ^2^	% Explained variability	Cumulative %
0.35276	0.12444	335.37	77.79	77.79
0.13503	0.01823	49.14	11.40	89.18
0.10108	0.01022	27.53	6.39	95.57
0.06651	0.00442	11.92	2.77	98.34
0.04745	0.00225	6.07	1.41	99.74
0.02027	0.00041	1.11	0.26	100.00

## Discussion

The wide range of helminths detected in the present study in reptiles living in sympatry with pets and the fact that many of these are parasitic and can infect companion animals (e.g., *D*. *acanthotetra*, *J*. *pasqualei*, *J. echinorhyncoides, Physaloptera* sp.) and humans (i.e., *Macracanthorhynchus* *hirudinaceus*, *Mesocestoides* *lineatus*) indicate the potential importance of reptiles as intermediate/paratenic hosts of parasitic helminths. Indeed, published data indicate that predation by pets could represent a transmission route for endoparasites [9].

The percentage of reptiles found to be infected by helminths (28.9%) is lower than that previously reported (i.e., 67–98%) [26, 28, 63], probably because the majority of those studies were focused on a single host species [24, 26, 28] or used different diagnostic methods (i.e., coprological techniques) [6, 64]. In addition, the number of individuals of some reptile species (i.e., *Hemidactylus turcicus*, *Hierophis carbonarius*, *Python molurus*) analyzed here was low, which likely led to underestimation of their helminth fauna. The high infection rates recorded here for *P. siculus* and* T*. *mauritanica* may be related to the fact that these two species were the most abundant, and especially so in dog shelters and urban and peri-urban areas. The detection of *M*. *hirudinaceus* in *P*. *siculus* is unprecedented and may be explained by the high prevalence of this zoonotic helminth in wild boar and intermediate hosts from the same area [65, 66]. Although little is known about the role of reptiles as paratenic hosts of *M*. *hirudinaceus* [67], they have been implicated in the life cycle of other zoonotic species of *Macracanthorhynchus* (i.e., *Macracanthorhynchus ingens* and *Macracanthorhynchus cutulinus*) [4, 68, 69]. To date, human cases of accidental infection through the ingestion of intermediate/paratenic hosts parasitized by *M*. *hirudinaceus* have been described mostly from other countries (i.e., Morocco, Argentina, Iran, and Tunisia) [66].

The detection of *J*. *echinorhyncoides*, *J*. *pasqualei* and *D*. *acanthotetra* in reptiles living in sympatry with companion animals highlights a potential link between pets and infection with these parasites through ingestion of these small vertebrates. Indeed, these cestodes have been reported from domestic [54, 70, 71] and feral cats [72, 73] and, to a lesser extent, from dogs [54, 74]. *M*. *lineatus* is known to circulate in wild and domestic carnivores in Italy [75–77]. Its detection in the present study from dog shelters shows the importance of treating pets with anthelmintics. Indeed, *Mesocestoides* sp. infections in dogs can become severe, e.g. with the development of peritoneal larval cestodiasis, where larvae penetrate the host’s intestinal wall and cause potentially life-threatening peritonitis [78]. Although no human cases of infection with *Mesocestoides* spp. have been recorded in Europe, these species may be of zoonotic relevance due to the incidental ingestion of their intermediate hosts [9, 79]. Among the nematodes found here, the only species of veterinary relevance was *Physaloptera* sp., a well-known agent causing vomiting and weight loss in cats and dogs [80–82]. Although its occurrence here is lower than that previously reported [25, 45], its detection confirms the circulation in southern Italy [83, 84], which should not be overlooked since *Physaloptera* spp. can cause severe clinical signs in cats, including gastric erosions and marked catarrhal gastritis [85, 86]. A massive infection by *Physaloptera* sp. was described in a young cat from a shelter in southern Louisiana [82], which indicates the importance of parasite control strategies for cats kept in these facilities.

Overall, the results discussed above suggest cats as more susceptible than dogs to helminth infections through the ingestion of reptiles, as they are more likely to show predatory behavior and can adapt to many types of environments [87]. In an Italian study, 21% of the prey of cats, which are considered excellent hunters, were reptiles [87, 88], and the preying of cats on these animals may support the trophic transmission of parasitic diseases [9]. The association between the lizards *P. siculus *and cestodes, along with *T*. *mauritanica* geckos and untreated pets, highlights the possible role of reptiles as sources of helminth infections and potentially zoonotic parasites.

## Conclusions

Synanthropic geckos and lizards represent an interface between wildlife and domestic settings. Encounters between these small vertebrates, companion animals and humans may lead to health issues, such as the transmission of parasites through predation of these small animals by dogs and cats. The results of the present study highlight the presence of helminth parasites in squamate reptiles that could be transmitted to companion animals (e.g.,* J*. *pasqualei*, *J. echinorhyncoides, D*. *acanthotetra*; *Physaloptera* sp.) and humans (i.e., *Macracanthorhynchus* *hirudinaceus*, *Mesocestoides* *lineatus*) when they live in sympatry. In this context, reptiles may play a role in the maintenance of parasitic diseases of pets, which reinforce the importance of regular anthelmintic treatment of companion animals. Finally, whenever a gecko, a lizard or a snake is captured from the wild and brought into a domestic setting, it would be good practice to screen it for the presence of parasites to reduce the risk of pathogen introduction.

## Supplementary Information

**Additional file 1:** **Text S1.** Main morphological features used for the macroscopic diagnosis of each parasite taxon. **Figure S1** Larval stage of *Sphaerirostris picae*. **Figure S2** Larval stage of* Macracanthorhynchus hirudinaceus*. **Figure S3** Larval stage of* Diplopylidium acanthotetra*. **Figure S4** Larval stage of* Joyeuxiella echinorhyncoides*. **Figure S5** Larval stage of* Joyeuxiella pasqualei*. **Figure S6** Larval stage of* Mesocestoides lineatus*. **Figure S7** An adult of* Paradistomum mutabile*. **Figure S8** Larval stage of the nematode family Acuariidae. **Figure. S9** Larval stage of* Physaloptera* sp. **Figure S10** An adult of* Parapharygodon micipsae*. **Figure S11** An adult of* Moaciria icosiensis*. **Figure S12** An adult of* Spauligodon aloisei*. **Table S1** Morphometric measurements [length (*L*), width (*W*)] of helminths collected from reptiles (all measurements are given in micrometers)

**Additional file 2: Table S2** Eigenvectors of variables associated with dimensions 1 and 2 of the multiple correspondence analysis

## Data Availability

The data that support the findings of this study are available from the corresponding author upon reasonable request. All sequences generated in this study have been deposited in the GenBank database (*S*. *picae* OQ451868, *M*. *lineatus* OQ451939, *Physaloptera* sp. OQ466450).

## Abbreviations

DNA: Deoxyribonucleic acid
BLAST: Basic Local Alignment Search Tool
CI: Confidence interval
MCA: Multiple correspondence analysis
PCR: Polymerase chain reaction
µm: Micrometer
cm: Centimeter

## References

[1] Bomford M, Kraus F, Barry SC, Lawrence E (2009). Predicting establishment success for alien reptiles and amphibians: a role for climate matching. Biol Invasions.

[2] Shaw GH, Shaw GH (2018). End of *Cretaceous* extinction: the end of the dinosaurs. Great moments in the history of life.

[3] Wolfe AK, Bateman PW, Fleming PA (2018). Does urbanization influence the diet of a large snake?. Curr Zool.

[4] Mendoza-Roldan JA, Modry D, Otranto D (2020). Zoonotic parasites of reptiles: a crawling threat. Trends Parasitol.

[5] Copping J. Reptiles now more popular pets than dogs. The Telegraph. 2008;22. https://www.telegraph.co.uk/news/earth/3500882/Reptiles-now-more-popular-pets-than-dogs.html. Accessed 17 Feb 2021.

[6] Rinaldi L, Mihalca AD, Cirillo R, Maurelli MP, Montesano M, Capasso M (2012). Flotac can detect parasitic and pseudoparasitic elements in reptiles. Exp Parasitol.

[7] Schuppli CA, Fraser D, Bacon HJ (2014). Welfare of non-traditional pets. Rev Sci Tech.

[8] Rapporto Assalco—Zoomark. 2022. https://www.assalco.it/archivio10_documento-generico_0_1338.html. Accessed 17 Feb 2021.

[9] Mendoza Roldan JA, Otranto D (2023). Zoonotic parasites associated with predation by dogs and cats. Parasit Vectors.

[10] Olsen JL (1980). Life cycle of *Physaloptera rara* Hall and Wigdor, 1918 (Nematoda: Physalopteroidea) of canids and felids in definitive, intermediate, and paratenic hosts. Rev Iber Parasitol.

[11] Haralabidis ST, Papazachariadou MG, Koutinas AF, Rallis TS (1988). A survey on the prevalence of gastrointestinal parasites of dogs in the area of Thessaloniki. Greece J Helminthol.

[12] Fox SM, Burns J, Hawkins J (1988). Spirocercosis in dogs. Compend Contin Educ Pract Vet.

[13] Millán J, Casanova JC (2009). High prevalence of helminth parasites in feral cats in Majorca Island (Spain). Parasitol Res.

[14] Jeżewski W, Buńkowska-Gawlik K, Hildebrand J, Perec-Matysiak A, Laskowski Z (2013). Intermediate and paratenic hosts in the life cycle of *Aelurostrongylus abstrusus* in natural environment. Vet Parasitol.

[15] Nabavi R, Manouchehri Naeini K, Zebardast N, Hashemi H (2014). Epidemiological study of gastrointestinal helminthes of canids in Chaharmahal and Bakhtiari province of Iran. Iran J Parasitol.

[16] Berrilli E, Simbula G (2020). First molecular identification of the tapeworm *Mesocestoides litteratus* from an Italian wall lizard (*Podarcis siculus*). Infect Genet Evol.

[17] Wu T, Bowman DD (2020). Visceral *larval migrans* of *Toxocara canis* and *Toxocara cati* in non-canid and non-felid hosts. Adv Parasitol.

[18] McGarry J, Collins M, Baross K (2020). UK report of tapeworm *Mesocestoides litteratus*. Vet Rec.

[19] Galán-Puchades MT, Mas-Coma S, Valero MA, Fuentes MV (2021). First data on the helminth community of the smallest living mammal on earth, the Etruscan pygmy shrew, *Suncus etruscus* (Savi, 1822) (Eulipotyphla: Soricidae). Animals.

[20] Radhakrishnan S, Kurup SP, Banerjee PS (2009). Endoparasitism in captive wild-caught snakes indigenous to Kerala. India Zoo Biol.

[21] Sharpilo VP, Biserkov VV, Kostadinova A, Behnke JM, Kuzmin YI (2001). Helminths of the sand lizard, *Lacerta agilis* (Reptilia, Lacertidae), in the Palaearctic: faunal diversity and spatial patterns of variation in the composition and structure of component communities. Parasitology.

[22] Rugiero L (2004). Composition of the reptile communities in five urban protected areas of different isolation degrees. Herpetozoa.

[23] Roca V, Foufopoulos J, Valakos E, Pafilis P (2009). Parasitic infracommunities of the Aegean wall lizard *Podarcis erhardii* (Lacertidae, Sauria): isolation and impoverishment in small island populations. Amphib-Reptil.

[24] Santoro M, Aznar FJ, Mattiucci S, Kinsella JM, Pellegrino F, Cipriani P (2013). Parasite assemblages in the western whip snake *Hierophis viridiflavus carbonarius* (Colubridae) from southern Italy. J Helminthol.

[25] Rataj AV, Lindtner-Knific R, Vlahović K, Mavri U, Dovč A (2011). Parasites in pet reptiles. Acta Vet Scand.

[26] Incedogan S, Yildirimhan HS, Bursey CR (2014). Helminth parasites of the ocellated skink, *Chalcides ocellatus* (Forskal, 1775) (Scincidae) from Turkey. Comp Parasitol.

[27] Cervone M, Fichi G, Lami A, Lanza A, Damiani GM, Perrucci S (2016). Internal and external parasitic infections of pet reptiles in Italy. J Herpetol Med Surg.

[28] Birlik S, Sami Yildirimhan H, Ilgaz Ç, Kumlutaş Y (2018). Helminth fauna of spiny tailed lizard, *Darevskia Rudis* (Bedriaga, 1886) (Sauria: Lacertidae) from Turkey. Helminthologia.

[29] Schuster RK, Gorcea MA, Neculicioiu VS, Junie LM, Codrean AG, Junie LM (2020). Cestodes of the genera *Diplopylidium* and *Joyeuxiella* (Eucestoda: Dipylidiiae) a review of historical data, species inventory and geographical distribution. Sci Parasitol.

[30] Birlik S, Yıldırımhan HS, Sümer N, Ilgaz Ç, Kumlutaş Y, Güçlü Ö (2015). The helminth fauna of *Apathya cappadocica* (Werner, 1902) (Anatolian lizard) (Squamata: Lacertidae) from Turkey. Helminthologia.

[31] Bursey CR, Goldberg SR, Telford SR (2007). Gastrointestinal helminths of 14 species of lizards from Panama with descriptions of five new species. Comp Parasitol.

[32] Mendoza-Roldan JA, Colella V, Lia RP, Nguyen VL, Barros-Battesti DM, Iatta R, Dantas-Torres F, Otranto D (2019). *Borrelia burgdorferi* (sensu lato) in ectoparasites and reptiles in southern Italy. Parasit Vectors.

[33] Mendoza-Roldan JA, Ravindran Santhakumari Manoj R, Latrofa MS, Iatta R, Annoscia G, Lovreglio P (2021). Role of reptiles and associated arthropods in the epidemiology of rickettsioses: a One Health paradigm. PLOS Negl Trop Dis.

[34] Mendoza-Roldan JA, Latrofa MS, Tarallo VD, Manoj RR, Bezerra-Santos MA, Annoscia G (2022). *Leishmania* spp. in squamata reptiles from the Mediterranean Basin. Transbound Emerg Dis.

[35] Warren K (2014). Reptile euthanasia—no easy solution?. Pac Conserv Biol.

[36] Witenberg G (1932). On the cestode subfamily Dipylidiinae Stiles. Z Parasitenkd.

[37] Timon-David J, Timon-David P (1967). Recherches expérimentales sur le cycle vital de *Paradistomum mutabile* (Molin) parasite de la vésicule biliaire de *Lacerta muralis* (Laurenti). Ann Parasitol Hum Comp.

[38] Miller DM, Dunagan TT (1971). Studies on the rostellar hooks of *Macracanthorhynchus hirudinaceus* (Acanthocephala) from swine. Trans Am Microsc Soc.

[39] Rysavý B (1973). Cysticercoids of cestodes of the family Dipylididae (Mola, 1929) from Egyptian snakes. Folia Parasitol.

[40] Adamson ML (1981). *Parapharyngodon osteopili* n. sp. (Pharyngodonidae: Oxyuroidea) and a revision of *Parapharyngodon* and *Thelandros*. Syst Parasitol.

[41] Jones A (1983). A revision of the cestode genus *Joyeuxiella* Fuhrmann, 1935 (Dilepididae: Dipylidiinae). Syst Parasitol.

[42] Casanova JC, Milazzo C, Ribas A, Cagnin M. *Spauligodon aloisei* n. sp. (Nematoda: Pharyngodonidae) parasite of *Podarcis sicula* (Reptilia: Lacertidae) from Italy. J Parasitol. 2003;89:577–9. doi: 10.1645/0022 3395(2003)089[0577:SANSNP]2.0.CO;2. 10.1645/0022-3395(2003)089[0577:SANSNP]2.0.CO;2 12880259

[43] Roca V (2003). A new genus of Dicrocoeliidae (Digenea) from the lizard *Gallotia atlantica* (Sauria: Lacertidae) from the Canary Islands (Spain). J Nat Hist.

[44] Richardson DJ (2005). Identification of cystacanths and adults of *Oligacanthorhynchus tortuosa*, *Macracanthorhynchus ingens*, and *Macracanthorhynchus hirudinaceus* based on probiscis and hook morphometrics. JAAS.

[45] Bursey C, Goldberg S, Kraus F. A new species of *Moaciria* (Nematoda, Heterakidae) and other helminths in the red Mawatta frog, *Hylophorbus* df. *rufescens* (Anura, Microhylidae) from Papua New Guinea. Acta Parasitol. 2007;52: 233–7.

[46] Gürelli G, Göçmen B, Çetin-Doğan T, Alpagut-Keskin N (1863). First record of* Mesocestoides* spp. Vaillant, Tetrathyridia (Cestoidea: Cyclophyllidea) in Anatolian lizard, *Anatololacerta danfordi* (Günther, 1876) in Turkey. North-West J Zool.

[47] Mašova S, Barus V, Hodová I, Koubek P, Koubková B (2009). Redescription of *Parapharyngodon micipsae* (Seurat 1917) (Nematoda, Pharyngodonidae) from the new host *Tarentola parvicarinata* Joger 1980 (Squamata, Gekkonidae) 1. Trop Zool.

[48] Amin OM, Heckmann RA, Halajian A, Eslami A (2010). Redescription of *Sphaerirostris picae* (Acanthocephala: Centrorhynchidae) from magpie, *Pica pica*, in northern Iran, with special reference to unusual receptacle structures and notes on histopathology. J Parasitol.

[49] Hrčkova G, Miterpáková M, O'Connor A, Šnábel V, Olson PD (2011). Molecular and morphological circumscription of *Mesocestoides* tapeworms from red foxes (*Vulpes vulpes*) in central Europe. Parasitology.

[50] Cho SH, Kim TS, Kong Y, Na BK, Sohn WM (2013). Tetrathyridia of *Mesocestoides lineatus* in Chinese snakes and their adults recovered from experimental animals. Korean J Parasitol.

[51] Tanveer S, Ahad S, Chishti MZ (2015). Morphological characterization of nematodes of the genera *Capillaria*, *Acuaria*, *Amidostomum*, *Streptocara*, *Heterakis*, and *Ascaridia* isolated from intestine and gizzard of domestic birds from different regions of the temperate Kashmir valley. J Parasit Dis.

[52] Kamimura K, Yonemitsu K, Maeda K, Sakaguchi S, Setsuda A, Varcasia A, Sato H (2018). An unexpected case of a Japanese wild boar (*Sus scrofa leucomystax*) infected with the giant thorny-headed worm (*Macracanthorhynchus hirudinaceus*) on the mainland of Japan (Honshu). Parasitol Res.

[53] Santana-Hernández KM, Orós J, Priestnall SL, Monzón-Argüello C, Rodríguez-Ponce E (2021). Parasitological findings in the invasive California kingsnake (*Lampropeltis californiae*) in Gran Canaria, Spain. Parasitology.

[54] Bezerra-Santos MA, Mendoza-Roldan JA, Lia RP, Annoscia G, Schuster R, Varcasia A (2022). Description of *Joyeuxiella pasqualei* (Cestoda: Dipylidiidae) from an Italian domestic dog, with a call for further research on its first intermediate host. Parasitology.

[55] Nascimento GF, Vieira FM, Gomes EC, Albinati ACL, Pereira LCM, Moura GJ (2022). Morphological description of infective larval stage of *Physaloptera* (Spirurida: Physalopteridae), and histological lesions in the paratenic host *Leptodactylus macrosternum* (Anura: Leptodactylidae) in Caatinga biome, Brazil. Rev Mex Biodivers.

[56] Skrjabin KI, Shikhobalova NP, Lagodovskaya EA. Oxyurata of animals and man. Part one. Oxyuroidea; 1974.

[57] Kearse M, Moir R, Wilson A, Stones-Havas S, Cheung M, Sturrock S (2012). Geneious basic: an integrated and extendable desktop software platform for the organization and analysis of sequence data. Bioinformatics.

[58] Vargas-Bello-Pérez E, Tajonar K, Foggi G, Mele M, Simitzis P, Mavrommatis A (2022). Consumer attitudes toward dairy products from sheep and goats: a cross-continental perspective. J Dairy Sci.

[59] Greenacre MJ, Greenacre MJ, Blasius J (2006). From simple to multiple correspondence analysis. Multiple correspondence analysis and related methods.

[60] George D, Mallery P. SPSS for windows step by step: a simple guide and reference 11.0 Update. 4th ed. Allyn Bacon; 2003.

[61] Sergeant ESG. Epitools epidemiological calculators. Ausvet Pty Ltd. 2018; http://epitools.ausvet.com.au. Accessed 18 Feb 2023.

[62] Taber KS (2018). The use of Cronbach’s alpha when developing and reporting research instruments in science education. Res Sci Educ.

[63] Jarulė V, Radziulis K, Urbanavičius M (2021). The comparison of helminth infections in pet snakes among amateur and professional snake keepers. Prof Stud Theory Pract.

[64] Papini R, Manetti C, Mancianti F (2011). Coprological survey in pet reptiles in Italy. Vet Rec.

[65] Migliore S, Puleio R, Gaglio G, Vicari D, Seminara S, Sicilia ER (2021). A neglected parasite: *Macracanthorhynchus hirudinaceus*, first report in feral pigs in a natural park of Sicily (southern Italy). Front Vet Sci.

[66] Dessì G, Cabras P, Mehmood N, Ahmed F, Porcu F, Veneziano V (2022). First molecular description of *Macracanthorhynchus hirudinaceus* in wild boars from Italy with pathomorphological and epidemiological insights. Parasitol Res.

[67] Kates KC (1943). Development of the swine thorn-headed worm, *Macracanthorhynchus hirudinaceus*, in its intermediate host. Am J Vet Res.

[68] Khokhlova IG (1986). Acanthocephalans of terrestrial vertebrates from the fauna of USSR.

[69] Hartnett EA, Léveillé AN, French SK, Clow KM, Shirose L, Jardine CM (2018). Prevalence, distribution, and risk factors associated with *Macracanthorhynchus ingens* infections in raccoons from Ontario, Canada. J Parasitol.

[70] Schuster RK, Thomas K, Sivakumar S, O’Donovan D (2009). The parasite fauna of stray domestic cats (*Felis catus*) in Dubai United Arab Emirates. Parasitol Res.

[71] Borji H, Razmi G, Ahmadi A, Karami H, Yaghfoori S, Abedi V (2011). A survey on endoparasites and ectoparasites of stray cats from Mashhad (Iran) and association with risk factors. J Parasit Dis.

[72] Lengy J, Steiman I, Steiman Y (1969). The current helmintofauna of stray dogs and cats in Israel. J Parasitol.

[73] Calvete C, Lucientes J, Castillo JA, Estrada R, Gracia MJ, Peribáñez MA (1998). Gastrointestinal helminth parasites in stray cats from the mid-Ebro Valley, Spain. Vet Parasitol.

[74] Dalimi A, Sattari A, Motamedi G (2006). A study on intestinal helminthes of dogs, foxes and jackals in the western part of Iran. Vet Parasitol.

[75] Bonfanti U, Bertazzolo W, Pagliaro L, Demarco B, Venco L, Casiraghi M (2004). Clinical, cytological and molecular evidence of *Mesocestoides* sp. infection in a dog from Italy. J Vet Med A Physiol Pathol Clin Med.

[76] Di Cerbo AR, Manfredi MT, Bregoli M, Milone NF, Cova M (2008). Wild carnivores as source of zoonotic helminths in north-eastern Italy. Helminthologia.

[77] Jabbar A, Papini R, Ferrini N, Gasser RB (2012). Use of a molecular approach for the definitive diagnosis of proliferative larval mesocestoidiasis in a cat. Infect Genet Evol.

[78] Carta S, Corda A, Tamponi C, Dessì G, Nonnis F, Tilocca L (2021). Clinical forms of peritoneal larval cestodiasis by *Mesocestoides* spp. in dogs: diagnosis, treatment and long term follow-up. Parasitol Res.

[79] Fuentes MV, Galan-Puchades MT, Malone JB (2003). Short report: a new case report of human *Mesocestoides* infection in the United States. Am J Trop Med Hyg.

[80] Soderman L, Harkin KR (2021). Gastric *Physaloptera* infection in 27 dogs (1997–2019). J Am Anim Hosp Assoc.

[81] de Macedo MRP, Zanet S, Bruno S, Tolosano A, Marucco F, Rossi L (2019). Gastrointestinal helminths of wolves (*Canis lupus* Linnaeus, 1758) in Piedmont, north-western Italy. J Helminthol.

[82] Lima JCMP, Del Piero F (2021). Severe concomitant *Physaloptera* sp., *Dirofilaria immitis*, *Toxocara cati*, *Dipylidium caninum*, *Ancylostoma* sp. and *Taenia taeniaeformis* infection in a cat. Pathogens.

[83] Milazzo C, de Bellocq JG, Cagnin M, Casanova JC, di Bella C, Feliu C (2003). Helminths and ectoparasites of *Rattus rattus* and *Mus musculus* from Sicily, Italy. Comp Parasitol.

[84] Santoro M, Tripepi M, Kinsella JM, Panebianco A, Mattiucci S (2010). Helminth infestation in birds of prey (Accipitriformes and Falconiformes) in southern Italy. Vet J.

[85] Naem S, Asadi R (2013). Ultrastructural characterization of male and female *Physaloptera rara* (Spirurida: Physalopteridae): feline stomach worms. Parasitol Res.

[86] Maharana BR, Gupta S, Gupta S, Ganguly A, Kumar B, Chandratre GA (2021). First report of molecular and phylogenetic analysis of *Physaloptera praeputialis* in naturally infected stray cats from India. Parasitol Res.

[87] Mori E, Menchetti M, Camporesi A, Cavigioli L, Tabarelli de Fatis K (2019). License to kill? Domestic cats affect a wide range of native fauna in a highly biodiverse Mediterranean country. Front Ecol Evol.

[88] Sogliani D, Mori E (2019). The fox and the cat: sometimes they do not agree. Mamm Biol.

[89] Bowles J, Blair D, McManus DP (1992). Genetic variants within the genus *Echinococcus* identified by mitochondrial DNA sequencing. Mol Biochem Parasitol.

[90] Littlewood DTJ, Waeschenbach A, Nikolov PN (2008). In search of mitochondrial markers for resolving the phylogeny of cyclophyllidean tapeworms (Platyhelminthes, Cestoda) a test study with Davaineidae. Acta Parasitol.

[91] Casiraghi M, Bain O, Guerrero R, Martin C, Pocacqua V, Gardner SL (2004). Mapping the presence of *Wolbachia pipientis* on the phylogeny of filarial nematodes: evidence for symbiont loss during evolution. Int J Parasitol.

[92] Patterson-Kane JC, Gibbons LM, Jefferies R, Morgan ER, Wenzlow N, Redrobe SP (2009). Pneumonia from *Angiostrongylus vasorum* infection in a red panda (*Ailurus fulgens fulgens*). J Vet Diagn Invest.

[93] Littlewood DTJ, Olson PD, Littlewood DTJ, Bray RA (2014). Small subunit rDNA and the Platyhelminthes: signal, noise, conflict and compromise. Interrelationships of the Platyhelminthes.

