# Supplementary material for: Squamata reptiles as a potential source of helminth infections when preyed on by companion animals
Source: Parasit Vectors. 2023 Jul 14;16:233. doi: 10.1186/s13071-023-05852-8 (PMC10349445; doi:10.1186/s13071-023-05852-8)
Supplement: Supplementary file 1 — Additional file 1: Text S1. Main morphological features used for the macroscopic diagnosis of each parasite taxon. Figure S1 Larval stage of Sphaerirostris picae. Figure S2 Larval stage of Macracanthorhynchus hirudinaceus. Figure S3 Larval stage of Diplopylidium acanthotetra. Figure S4 Larval stage of Joyeuxiella echinorhyncoides. Figure S5 Larval stage of Joyeuxiella pasqualei. Figure S6 Larval stage of Mesocestoides lineatus. Figure S7 An adult of Paradistomum mutabile. Figure S8 Larval stage of the nematode family Acuariidae. Figure. S9 Larval stage of Physaloptera sp. Figure S10 An adult of Parapharygodon micipsae. Figure S11 An adult of Moaciria icosiensis. Figure S12 An adult of Spauligodon aloisei. Table S1 Morphometric measurements [length (L), width (W)] of helminths collected from reptiles (all measurements are given in micrometers) [file 13071_2023_5852_MOESM1_ESM.docx]

**Main morphological features used for macroscopic diagnosis of each parasite taxa**

**Fig. S1** Larval stage of *Sphaerirostris picae*.

*
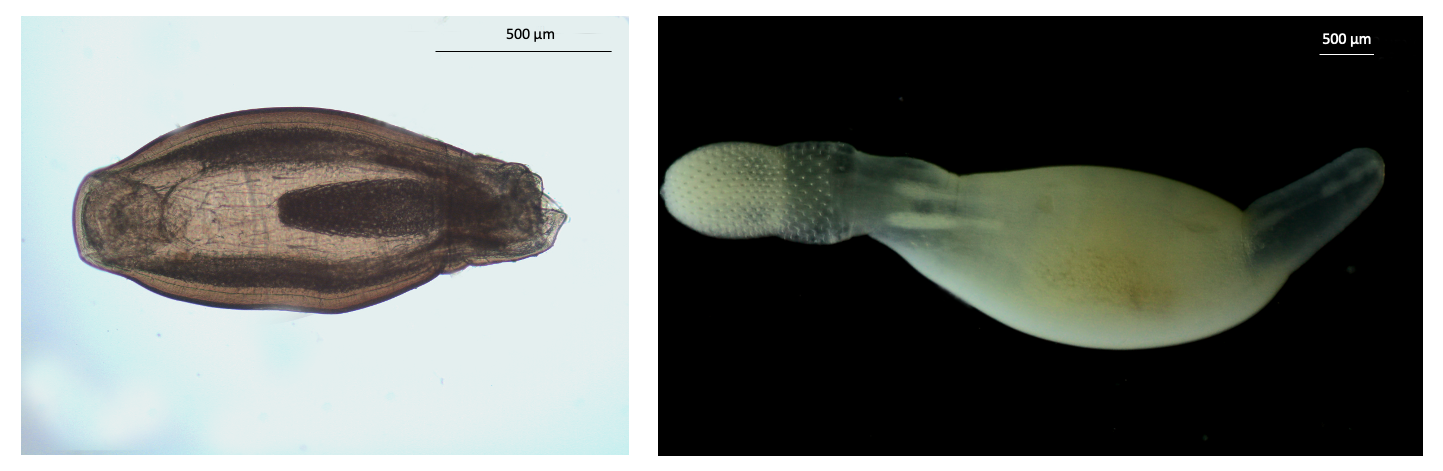
*

Fusiform and whitish cysts containing a cystacanth with trunk cylindrical, spindle shaped, gradually tapering toward both ends; proboscis provided with hook rows and separated in two parts by a constriction. Anterior proboscis ovoid, with hook length increasing apically. Posterior proboscis cylindrical with more widely spaced armature. Neck short and lemnisci visible (Table S1a) [48].

*
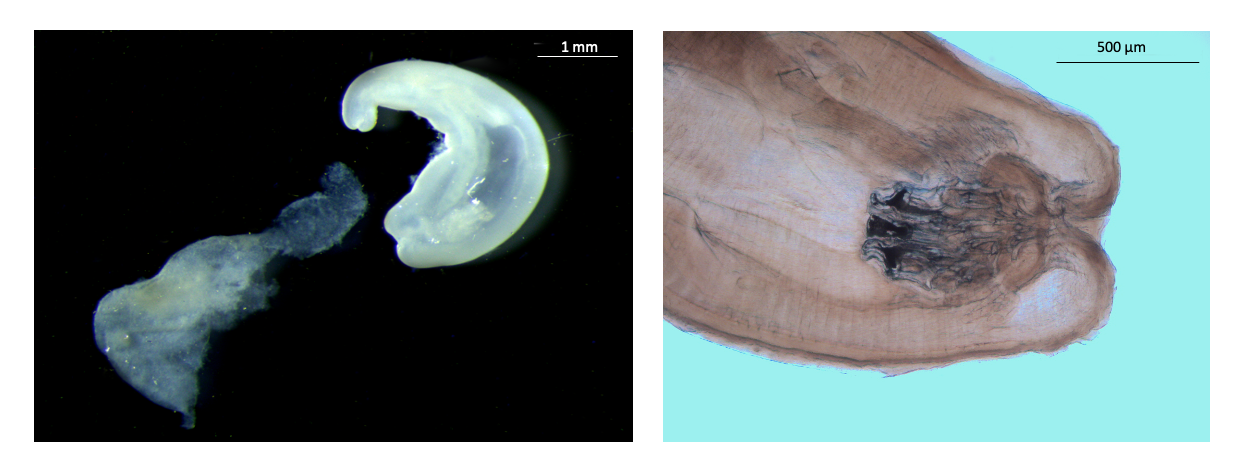
***Fig. S2** Larval stage of *Macracanthorhynchus hirudinaceus*.

Spherical cyst containing a cystacanth provided with thick wall and cylindrical proboscis bearing hooks in circular rows. Length of hooks increasing apically, with the longest one measuring 276.5 µm (Table S1a) [38, 44, 52].

**Fig. S3** Larval stage of *Diplopylidium acanthotetra*
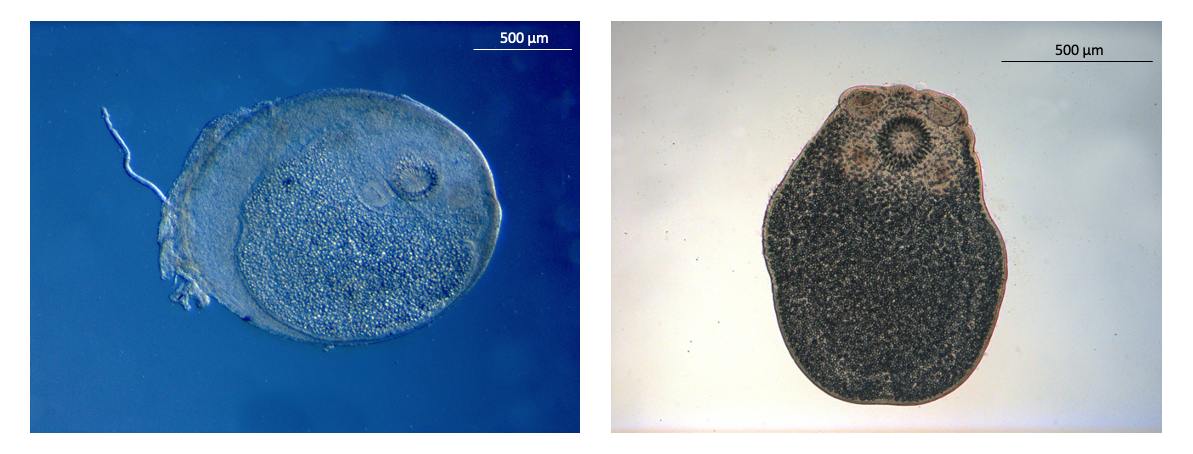
.

Single, multiple (in clusters) or merging cysts of spherical shape. Cysticercoid characterized by numerous calcareous corpuscles and circular rostellum, armed with hooks arranged in four circles (Table S1b) [29, 36, 39, 53].

**Fig. S4** Larval stage of *Joyeuxiella echinorhyncoides*.


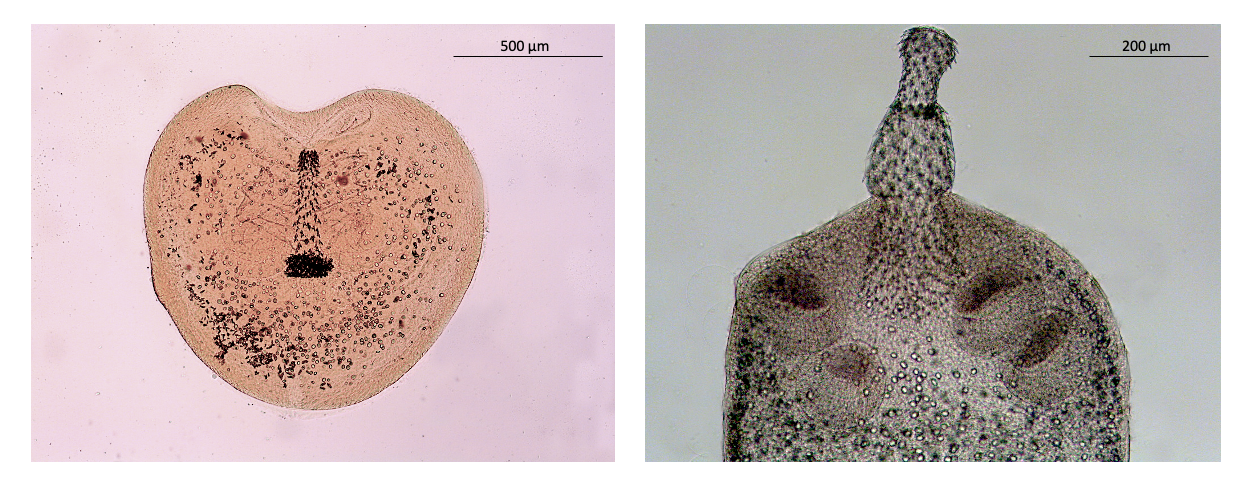


Single, multiple or merging cysts of spherical shape, containing a cysticercoid (more than one in case of merging cysts) heart-shaped characterized by tubular rostellum armed with hooks over the entire length, including the base invaginated (Table S1b) [29, 41].

*
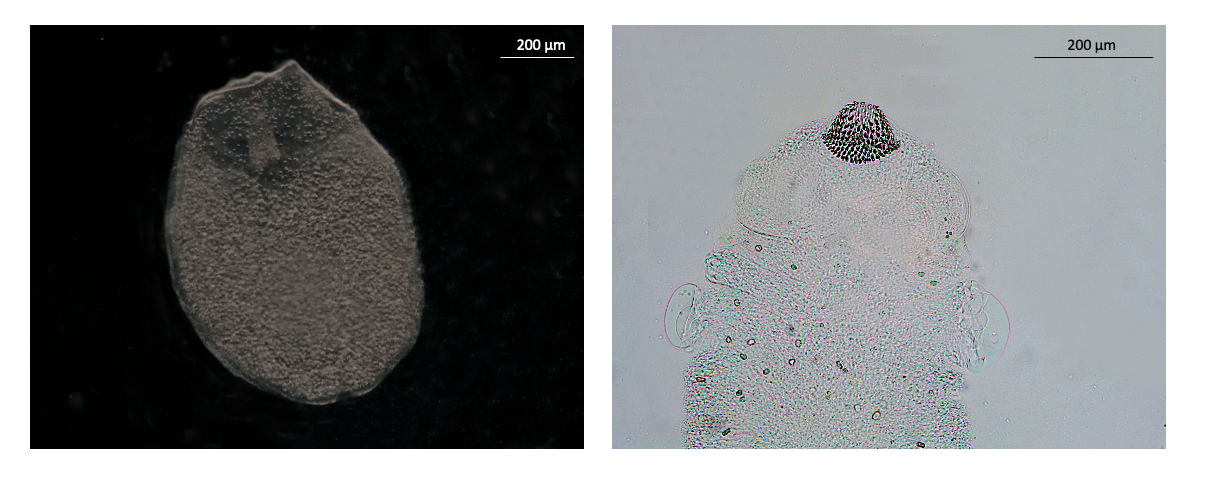
***Fig. S5** Larval stage of *Joyeuxiella pasqualei*.

Spherical cyst containing a cysticercoid characterized by conical rostellum, armed with several

alternating circles of thorn-shaped hooks (Table S1b) [29, 41, 54].

*
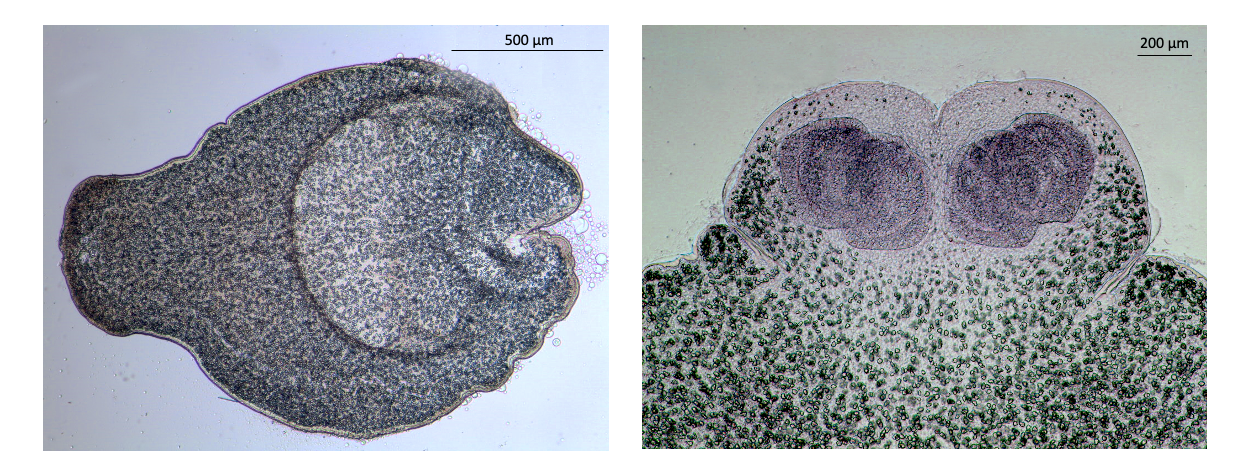
***Fig. S6** Larval stage of *Mesocestoides lineatus*.

Single or multiple spherical cysts containing a flattened and non-segmented tetrathynidium of pleomorphic shape (mostly longitudinally elongated), characterized by lacking rostellum and the presence of four visible suckers (Table S1b) [16, 46, 50].

*
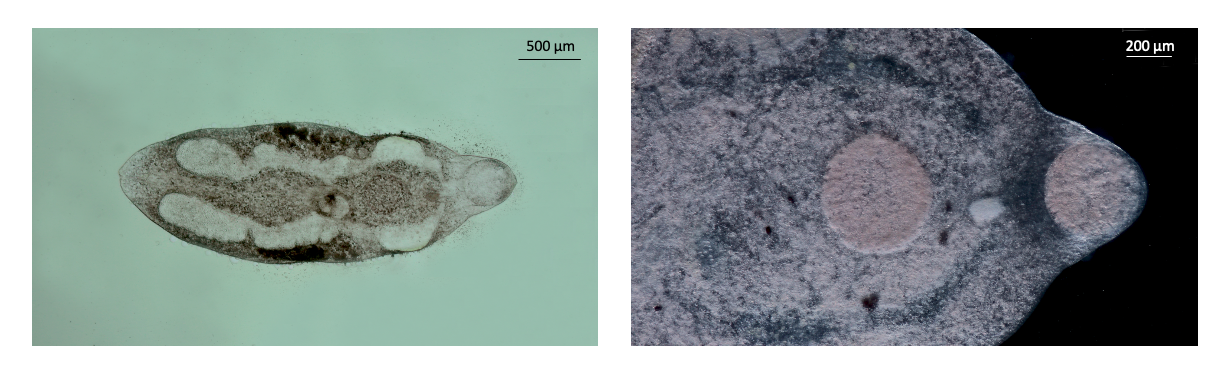
***Fig. S7** Adult of *Paradistomum mutabile*.

Adult digenea (4854-2525 x 2304-831µm, based on four specimens) characterized by clearly visible oral (412-260 µm wide) and ventral (602-274 µm wide) suckers, branched tubular uterus containing eggs of 52-45 µm length, a pair of branched vitellogen glands and intestinal caeca extend into posterior quarter of the body [37, 43].


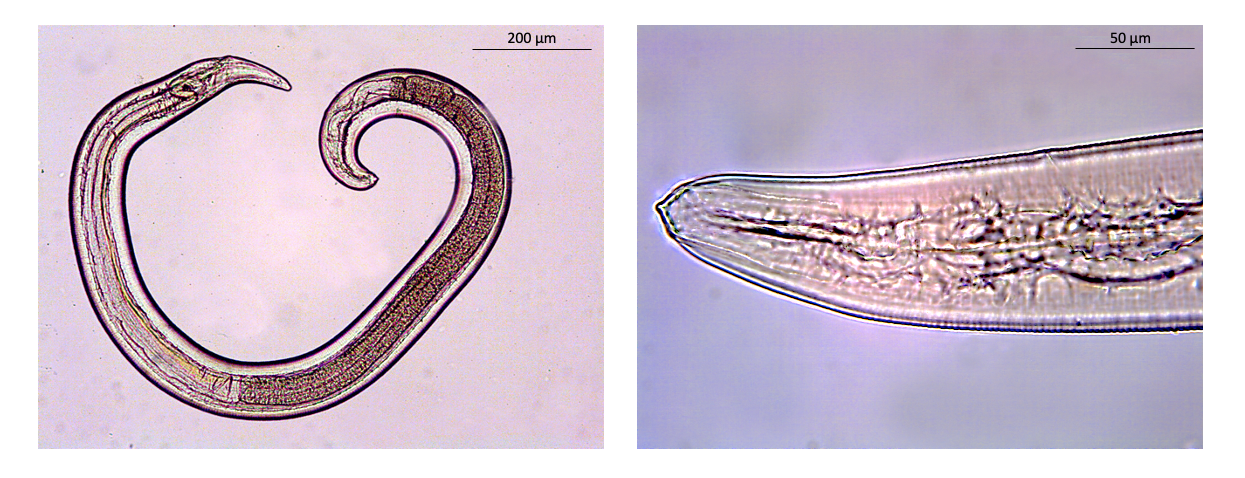
**Fig. S8** Nematode larval stage of the Family *Acuariidae*.

Spherical cyst containing a third-stage larvae spiralized, characterized by cephalic region with triangular lips and lateral cordons extending from the anterior end (Table S1c) [51].

**Fig. S9** Larval stage of *Physaloptera* sp..
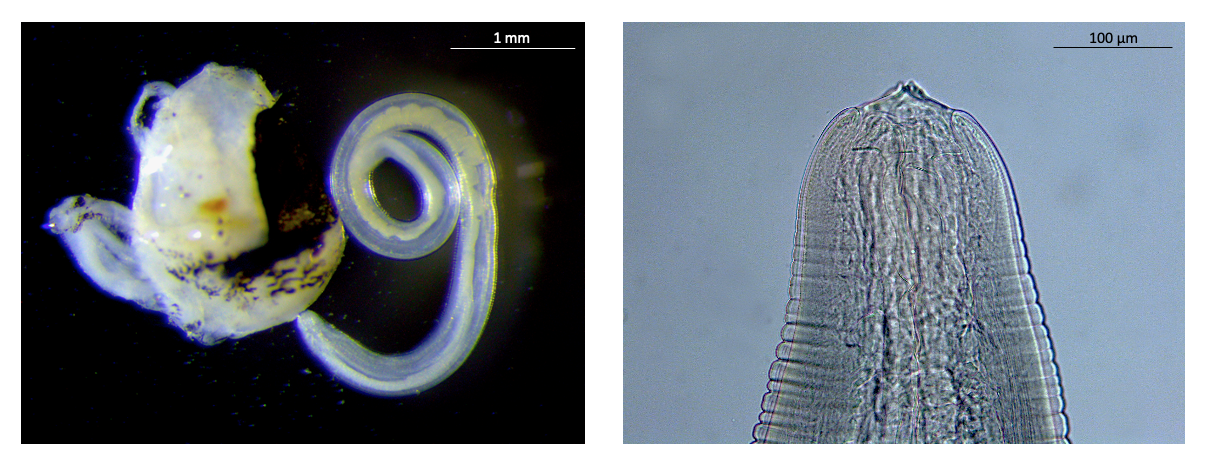


Spherical cyst containing a spiralized third-stage larvae characterized by thin and smooth cuticle and long muscular-glandular oesophagus. The cephalic region presents a collarette and the rounded anterior end consists of two well-developed lateral pseudolabia (Table S1c) [55].


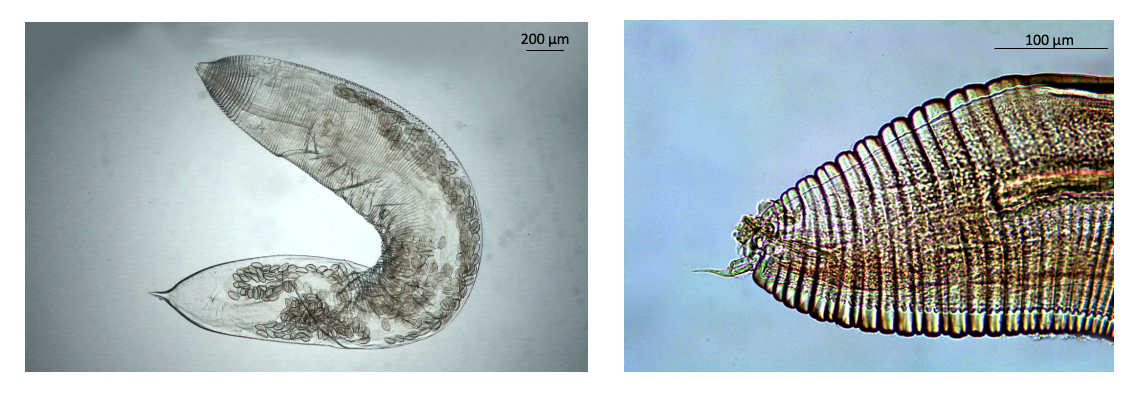
**Fig. S10** Adult of *Parapharyngodon micipsae*.

General - Small nematode, light-colored, fusiform and cuticle with distinct transverse striations.

Male - Body truncated posteriorly with lateral alae well developed. Three pairs of cloacal papillae and one on the tail. Spicule well sclerotized, sharp at the end.

Female - Body with cuticle annulations, anterior end bluntly pointed, posterior end tapered to fine spike. Oesophageal isthmus long and wide, as well as the bulbus (Table S1d) [40, 47, 56].

**Fig. S11** Adult of *Moaciria icosiensis*
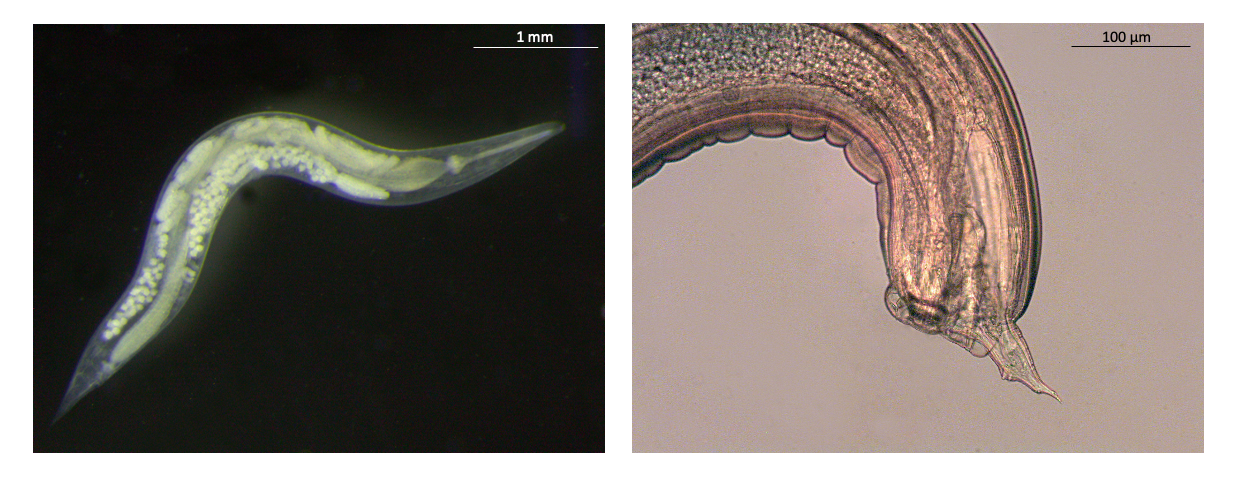
.

General - Small nematode with oesophagus divisible into anterior pharynx, corpus and posterior valved bulb.

Male - Body with a pair of equal spicules, characterized by proximal end truncate and distal tapered, ending in a sharp tip. Gubernaculum of arrow shape and well-developed

Female - Body stout characterized by thick cuticle and fine transverse striations. Vulva provided with minute vulvar lips, slightly protruding, just anterior to mid-length of body. Amphidelphic uterus. Tail thin and tapering to a fine point. Eggs numerous and smooth-shelled (Table S1e) [45, 56].

**Fig. S12** Adult of *Spauligodon aloisei*.
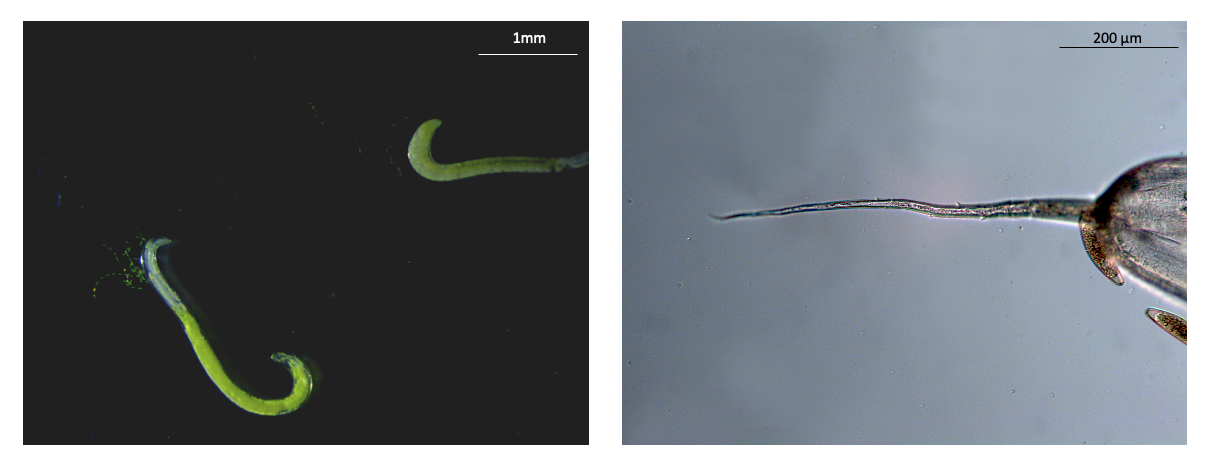


General - Small nematode of fusiform shape. Body with cuticle fine cross-striations and filiform tail with spines.

Female (single gender recorded) - Body (6500-5400 µm, based on two specimens) tapering anteriorly and posteriorly cylindrical. Esophagus (including bulb) length of 480-420µm; eggs (130-110µm) with truncated ends and tail length of 650-630 µm [42, 56].

**Table S1** Morphometric measurements (L: length; W: width) of helminths collected from reptiles (all measurements are given in micrometer).

| **1a)** Acanthocephalan larval stages. | | |  | | | |  | | |  | | | | | |  | | |  | |  | |  | |  | |  | |  | |  |
| --- | --- | --- | --- | --- | --- | --- | --- | --- | --- | --- | --- | --- | --- | --- | --- | --- | --- | --- | --- | --- | --- | --- | --- | --- | --- | --- | --- | --- | --- | --- | --- |
| **Species (n)** | **Cyst (L x W)** | | | **Cystacanth (L x W)** | | | | | **Proboscis (L)** | | | | | |  |  | | |  | |  | |  | |  | |  | |  | |  |
| *S*. *picae* (11) | 2450-1064 x 940-508 | | | 2122-1239 x 823-480 | | | | | 809-520 | | | | | |  |  | | |  | |  | |  | |  | |  | |  | |  |
| *M. hirudinaceus* (1) | 2205 x 1703 | | | 4180 x 2384 | | | | | 700 | | | | | |  |  | | |  | |  | |  | |  | |  | |  | |  |
|  |  | |  | | | | |  | | | |  | | | |  | | |  | |  | |  | |  | |  | |  | |  |
| **1b)** Cestode larval stages. | | |  | | | | |  | | | |  | | | |  | | |  | |  | |  | |  | |  | |  | |  |
| **Species (n)** | | **Cyst (L x W)** | | | | **Cysticercoid (L x W)** | | | | | **Rostellum (W)** | | | **Rostellum (L)** | | | |  | |  | |  | |  | |  | |  | |  | |
| *D. acanthotetra* (8) | | 2749-1200 x 2368-1500 | | | | 2600-936 x 1924-803 | | | | | 346-182 | | | - | | | |  | |  | |  | |  | |  | |  | |  | |
| *J. echinorhyncoides* (7) | | 2300-1053 x 1600-1019 | | | | 1700-810 x 988-603 | | | | | - | | | 503-286 | | | |  | |  | |  | |  | |  | |  | |  | |
| *J*. *pasqualei* (6) | | 2000-800 x 1450-900 | | | | 2300-780 x 1340-703 | | | | | - | | | 180-110 | | | |  | |  | |  | |  | |  | |  | |  | |
| *M*. *lineatus* (5) | | 791-523 x 615-431 | | | | 1619-1150 x 1345-848 | | | | | - | | | - | | | |  | |  | |  | |  | |  | |  | |  | |
|  |  | |  | | | | |  | | | |  | | | | |  | |  | |  | |  | |  | |  | |  | |  |
|  |  | |  | | | |  | | | | |  | | | |  | | |  | |  | |  | |  | |  | |  | |  |
| **1c)** Nematode third-stage larvae (total body length: TBL; muscular oesophagus length: MOL; glandular oesophagus length: GOL;  tail length: TL). | | | | | | | | | | | | | | | | | | | | | | | | | | |  | |  | |  |
| **Genera (n)** | **Cyst (L x W)** | | | | **TBL** | | **MOL** | | | **GOL** | | | **TL** | | | | | |  | |  | |  | |  | |  | |  | |  |
| Acuariidae specimens (5) | 1200-451 x 581-486 | | | | 2717-2100 | | 355-200 | | | 1200-800 | | | 120-101 | | | | | |  | |  | |  | |  | |  | |  | |  |
| *Physaloptera* sp. (4) | 3500-2000 x 3340-1600 | | | | 8800-7266 | | 383-280 | | | 2300-1900 | | | 230-190 | | | | | |  | |  | |  | |  | |  | |  | |  |
|  |  | |  | | | |  | | |  | | | | | |  | | |  | |  | |  | |  | |  | |  | |  |
| **1d)** Adult nematode *Parapharyngodon micipsae* (total body length: TBL; oesophagus + bulbus length: OBL; spicule length: SL;  anus-tail length: ATL; egg length: EL) | | | | | | | | | | | | | | | | | | | | | | | | | | | | | | |  |
| **Genders (n)** | **TBL** | | **OBL** | | | | **SL** | | | **ATL** | | | | | | **EL** | | |  | |  | |  | |  | |  | |  | |  |
| Males (2) | 2931-1630 | | 658-390 | | | | 80-60 | | | - | | | | | | - | | |  | |  | |  | |  | |  | |  | |  |
| Females (2) | 4700-4500 | | 1216-1193 | | | | - | | | 500-414 | | | | | | 89-80 | | |  | |  | |  | |  | |  | |  | |  |
|  |  | |  | | | |  | | |  | | | | | |  | | |  | |  | |  | |  | |  | |  | |  |
| **1e)** Adult nematode *Moaciria icosiensis* (total body length: TBL; oesophagus + bulbus length: OBL; spicule length: SL;  anus-tail length: ATL; egg length: EL) | | | | | | | | | | | | | | | | | | | | | | | | | | | | |  | |  |
|  |  | |  | | | |  | | |  | | | | | |  | | |  | |  | |  | |  | |  | |  | |  |
| **Genders (n)** | **TBL** | | **OBL** | | | | **SL** | | | **ATL** | | | | | | **EL** | | |  | |  | |  | |  | |  | |  | |  |
| Males (4) | 4500-4178 | | 955-777 | | | | 315-290 | | | - | | | | | | - | | |  | |  | |  | |  | |  | |  | |  |
| Females (1) | 5600 | | 900 | | | | - | | | 550 | | | | | | 63-87 | | |  | |  | |  | |  | |  | |  | |  |
|  |  | |  | | | |  | | |  | | | | | |  | | |  | |  | |  | |  | |  | |  | |  |
