# Supplementary material for: Squamata reptiles as a potential source of helminth infections when preyed on by companion animals
Source: Parasit Vectors. 2023 Jul 14;16:233. doi: 10.1186/s13071-023-05852-8 (PMC10349445; doi:10.1186/s13071-023-05852-8)
Supplement: Supplementary file 2 — Additional file 2: Table S2 Eigenvectors of variables associated with dimensions 1 and 2 of the multiple correspondence analysis [file 13071_2023_5852_MOESM2_ESM.docx]

**Table S2** Eigenvectors of variables associated to dimension 1 and 2 of the multiple correspondence analysis.

| **Factors** | **Category** | **Dimension 1** | **Dimension 2** | **Dimension 3** |
| --- | --- | --- | --- | --- |
| Captured dead or alive | alive | -0,0209 | -0,092 | -0,0338 |
| Captured dead or alive | dead | 0,3203 | 1,417 | 0,5188 |
| Region | Apulia | 0,5319 | 0,18 | -0,0671 |
| Region | Basilicata | 0,7378 | -0,04 | -0,0392 |
| Region | Calabria | 0,6194 | -0,374 | 0,5716 |
| Region | Sicily | -0,8016 | -0,101 | -0,0592 |
| Characteristics of the area | dog shelter | 0,6855 | -0,151 | 0,1586 |
| Characteristics of the area | household | 0,5465 | -0,015 | -0,1741 |
| Characteristics of the area | Peri urban | 0,4117 | 0,004 | -0,2692 |
| Characteristics of the area | regional park | 0,8472 | -0,055 | 0,0379 |
| Characteristics of the area | Rural | -0,3557 | 0,106 | -0,8102 |
| Characteristics of the area | urban | -0,8297 | 0,109 | 0,0717 |
| Presence of pets | no pets | 0,6066 | -0,023 | -0,1317 |
| Presence of pets | pets | -0,0258 | 0,001 | 0,0056 |
| Insecticides/repellents | insecticides/repellents | 0,6676 | -0,131 | 0,1096 |
| Insecticides/repellents | no insecticides/repellents | -0,3483 | 0,068 | -0,0572 |
| Anthelmintics | anthelmintics | 0,5465 | -0,015 | -0,1741 |
| Anthelmintics | no anthelmintics | -0,0281 | 0,001 | 0,009 |
| Cysts | cysts | 0,3665 | 0,103 | -0,1351 |
| Cysts | no cysts | -0,2658 | -0,075 | 0,098 |
| Acanthocephala | Acanthocephala | 0,3845 | -0,038 | -0,1489 |
| Acanthocephala | no Acanthocephala | -0,0578 | 0,006 | 0,0224 |
| Nematoda | Nematoda | -0,3272 | 0,147 | 0,0302 |
| Nematoda | no Nematoda | 0,0372 | -0,017 | -0,0034 |
| Cestoda | Cestoda | 0,4511 | 0,206 | -0,1396 |
| Cestoda | no Cestoda | -0,0653 | -0,03 | 0,0202 |
| Digenea | Digenea | 0,2931 | -0,048 | -0,1185 |
| Digenea | no Digenea | -0,0112 | 0,002 | 0,0045 |
